# A case of gastrointestinal tuberculosis in a 3-mo-old infant with profound immunodeficiency

**DOI:** 10.70962/jhi.20250247

**Published:** 2026-05-21

**Authors:** Christine Karanja-Chege, David Mwakisha Mwashigadi, Joseph Mbuthia, Peter Ngwatu, Jeremiah Kamwetu, Jacquie Oliwa

**Affiliations:** 1 Kenyatta University, Nairobi, Kenya; 2 Gertrude’s Children’s Hospital, Nairobi, Kenya; 3 Kenyatta National Hospital, Nairobi, Kenya; 4 Health Services Unit, KEMRI-Wellcome Trust Research Programme, Nairobi, Kenya; 5 Department of Public Health, Institute of Tropical Medicine, Antwerp, Belgium

## Abstract

Gastrointestinal tract tuberculosis (GIT-TB) is a rare form of TB, but one that poses a serious challenge in diagnosis and management, particularly in the setting of severe immunosuppression. We present a case of intestinal TB in a 3-mo-old immunosuppressed infant whose chief complaints were progressive abdominal distension and failure to thrive with features of intestinal obstruction and a diagnosis of GIT-TB confirmed on histology and culture. The resulting serious complications despite surgery included short bowel syndrome, enterocutaneous fistulae, and cholestasis necessitating the use of total parenteral nutrition and a modified parenteral TB treatment regimen. During the 2-mo stay in hospital, the patient developed irreversible liver failure and azotemia, eventually succumbing to these complications. This case highlights the importance of early recognition and treatment of GIT-TB to prevent adverse outcomes. Further, there is a need to investigate for immunodeficiency in such presentations, particularly in young infants. The challenges encountered by the clinicians underscore the importance of the development of World Health Organization guidelines on the specific management of GIT-TB.

## Teaser

Karanja-Chege et al. describe a rare case of gastrointestinal tuberculosis in a severely immunosuppressed three-month-old infant presenting with intestinal obstruction in Kenya. Despite surgery and intensive care, life-threatening complications develop. The report underscores the diagnostic challenges, need for early recognition, immunodeficiency evaluation, and clearer management guidance for this severe condition.

## Introduction

Tuberculosis (TB) is one of the leading causes of morbidity and mortality from infectious disease worldwide. In 2023, an estimated 10.8 million TB cases were reported globally, resulting in 1.25 million deaths. More than 80% of this burden is from low-income countries including Kenya (1). Childhood TB, which contributes ∼10% to the overall burden (1), poses a special challenge in diagnosis. The World Health Organization (WHO) screening tool (2) currently focuses only on pulmonary TB (3) and is therefore not helpful in diagnosing extrapulmonary TB forms.

Extrapulmonary TB includes abdominal TB, which is relatively rare, contributing to <3% of all TB. This TB form is characterized by involvement of the solid abdominal organs, the peritoneum, mesenteric lymph nodes, and/or the gastrointestinal tract (GIT) (4). The route of infection of the gut is predominantly by ingestion of *Mycobacteria tuberculosis* (MTB), with either spread occurring hematogenously or through contiguous spread from adjacent viscera. Gastrointestinal TB can affect the small bowel, with ulceration and scarring of the mucosal and submucosal tissue leading to inability to absorb nutrients and drugs, including conventional oral anti-TB medications (5, 6). The resulting short bowel phenomenon, which may make total parenteral nutrition (TPN) necessary, is commonly associated with cholestasis. Parenteral nutrition further worsens the already existing cholestasis and may eventually lead to end-stage liver disease (7). In the absence of established parenteral TB treatment protocols, clinicians have had to rely on evidence derived from case reports (8).

Young infants, with their immature immune system, are at a higher risk of infections including disseminated TB. Extrapulmonary or disseminated TB forms in this age group may, however, point to T cell and/or B cell deficiencies, with a compromised ability to mount an effective immune response to MTB (9).

We report a case of gastrointestinal TB in a 3-mo-old infant with an underlying unclassified immunodeficiency, who presented a diagnostic and treatment challenge to us. By sharing this case, including the modified treatment regimen we used and the complications that eventually led to the patient’s demise, we hope to highlight the need to come up with parenteral TB treatment guidelines for GIT-TB.

## Results

Serial hematological data are summarized in Table 1, denoting progressive derangement of most of the parameters. The hemoglobin levels over time dropped to a nadir of 6.7 g/dl, and there was the development of severe thrombocytopenia with a platelet count of 4 c/μl on the day of the child’s demise.

**Table 1.** Serial hemogram reports

Parameter/Date	10/11/24	17/11/24	16/12/24	23/12/24	30/12/24	8/1/25
WBC (c/μl)	20.46	15.3	3.81	9.69	5.69	7.15
Neutrophils (%)	52.3	85.6	46.8	69.20	74.70	85.20
Lymphocytes (%)	18.8	9.7	34.10	4.10	6.50	2.70
HB (g/dl)	7.5	9.3	8.6	9.2	8.60	6.70
MCV (fl)	79.5	78.5	83.3	84.9	85.20	83.50
Platelets (c/μl)	369	42	59	3	2	4

The coagulation profile as depicted by the INR rose over time, indicative of declining synthetic capacity of the liver with the progression to hepatic failure (Table 2).

**Table 2.** Coagulation profile

Parameter/Date	10/11/24	16/11/24	17/12/24	26/12/24	6/1/25
INR	1.8	1.5	1.4	1.4	2.1

Initial biochemistry tests revealed hypoglycemia of 3.3 mmol/L with normal thyroid function (TSH 3.88 μIU/ml, free T3 2.17 pg/ml, free T4 1.32 ng/dl), while the acute phase reactants (CRP and procalcitonin) showed a variable trend, consistent with severe infection and inflammation as shown in Table 3.

**Table 3.** CRP and PCT

Parameter/Date	10/11/24	17/11/24	12/12/24	2/1/25	6/1/25
CRP (mg/L)	116.11	209.91	5.94	47.10	57.24
PCT (ng/ml)	2.58	11.22	0.14	47.82	37.11

PCT, procalcitonin.

The renal (Table 4) and liver (Table 5) function tests were initially normal, but over time, there was the eventual development of azotemia and liver failure, respectively.

**Table 4.** Renal function tests

Parameter/Date	10/11/24	15/11/24	13/12/24	30/12/24	6/1/25
Urea (mmol/L)	6.07	2.32	3.10	7.31	14.23
Creatinine (mmol/L)	31	29	15	<18	<18
Sodium (mmol/L)	135.4	143	133.03	133.46	132.34
Potassium (mmol/L)	3.38	4.62	4.4	3.41	3.67

**Table 5.** Liver function tests

Parameter/Date	10/11/24	17/11/24	9/12/24	16/12/24	30/12/24	6/1/25
Total bilirubin (μmol/L)	15	17.6	18.6	38.5	481.3	452.4
Direct bilirubin (μmol/L)	12.3	16.2	16	29.5	387.5	416.0
ɣ-Glutamyl transferase (U/L)	162	131.7	266.3	312.3	141.3	50.2
Alanine aminotransferase (U/L)	7.7	15.7	28	20.9	16.6	15.1
Aspartate aminotransferase (U/L)	17.6	27.2	15.9	18.4	21.4	19.9
Albumin (g/L)	33.87	25	31.85	32.1	26.9	17.91

Tests for concomitant infections—HIV DNA PCR and malaria—were negative. We performed an immunological profile to screen for inborn errors of immunity by evaluating the lymphocyte subsets and immunoglobulin profile (Tables 6, 7, 8, and 9). The absolute counts of B, T, and natural killer (NK) cells were all low. The levels of the immunoglobulins IgA and IgM were within normal limits, while IgG was slightly elevated. IgE and IgD tests were not done.

**Table 6.** B lymphocyte subset

Parameter/Values	Observed value	Normal range
CD19 (%)	7.4	4.6–22.1
Absolute CD 19 count (cells/μl)	23.01	56.6–417.4

**Table 7.** NK lymphocyte subset

Parameter/values	Observed value	Normal range
CD3 cells (%)	85	51–77
Absolute CD3 count (cells/μl)	264	2,500–5,600
CD (16+56) %	7.9	3–14
Absolute CD (16+56) count (cells/μl)	24.57	170–830

**Table 8.** T lymphocyte subset

Parameter	Observed value	Normal range
CD45 absolute lymphocyte gated (/c.mm)	311	1,000–3,000
CD3 (T cells) (%)	84.68	51–77
CD3 (T cells) absolute (cells/μl)	264	2500–5,600
CD4 helper T cells (%)	76.67	35–56
CD4 helper T cells absolute (cells/μl)	239	1,800–4,000
CD8 suppressor T cells (%)	5.52	12–23
CD8 suppressor T cells absolute (cells/μl)	17	590–1,600
CD4/CD8 ratio	13.89	≥1.0

**Table 9.** Immunoglobulin profile (g/L)

Immunoglobulin (g/l)	Observed value	Normal levels
IgA	0.97	0.0–0.1
IgG	7.66	1.5–6.3
IgM	0.4	0.1–0.8

**Figure S1.**
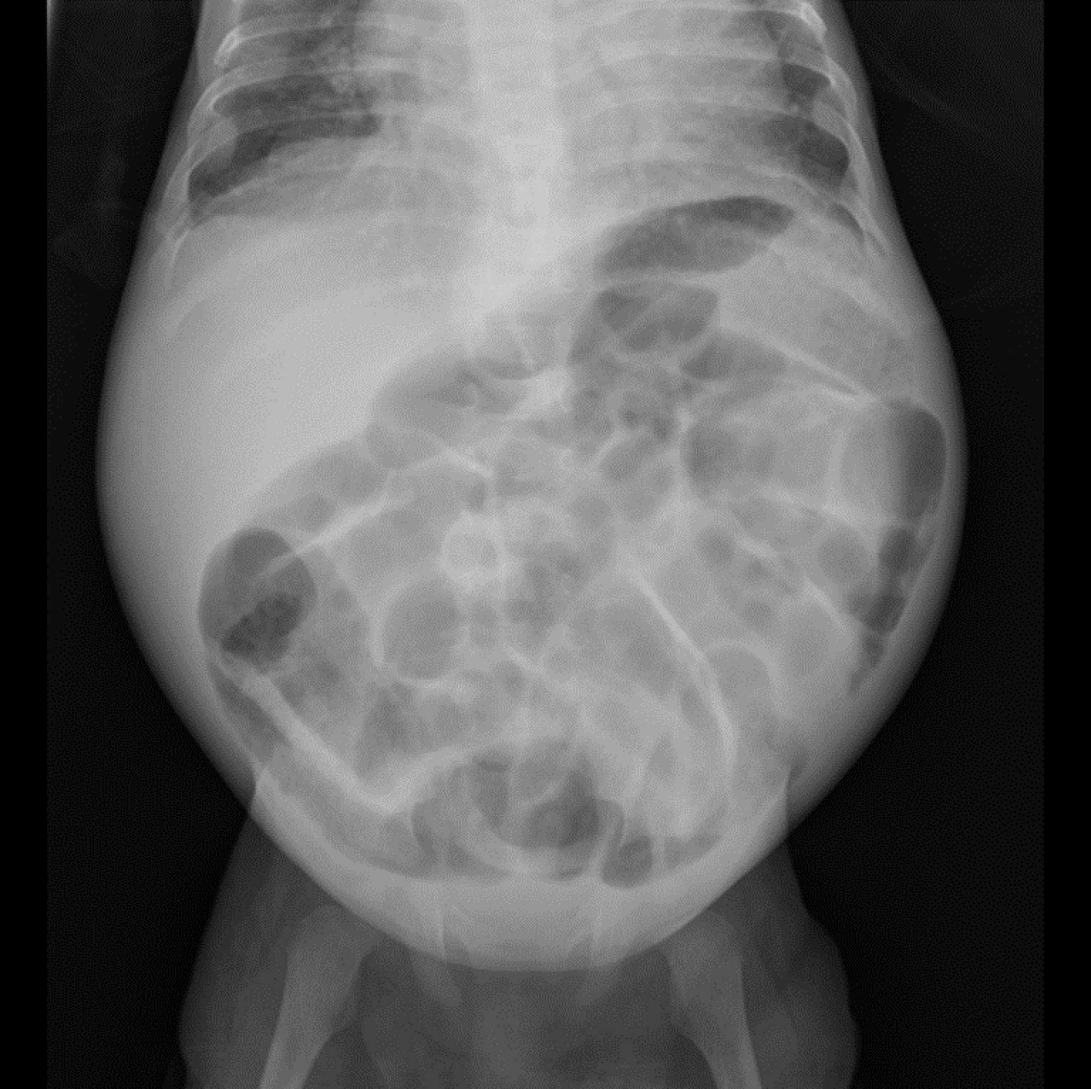
Inflamed bowel loops with gaseous distention.

A plain abdominal x ray done at admission demonstrated bowel loops distended with gas and extensive inflammation of the walls (Fig. S1).

**Figure S2.**
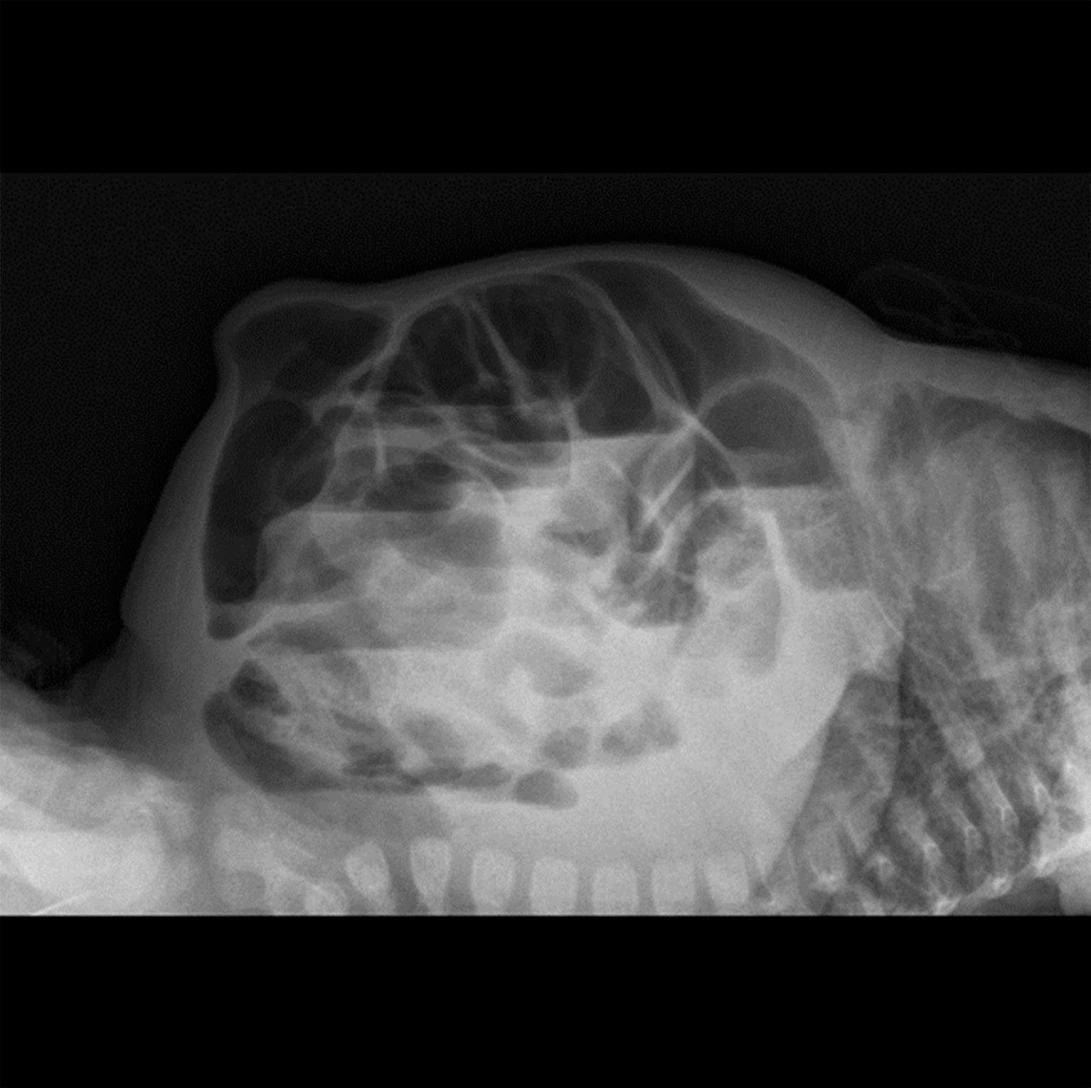
Dilated bowel loops with air-fluid levels.

A repeat abdominal x ray was done 2 days after a poor response to attempted gut decompression, which was done by nasogastric tube insertion, nil-by-mouth orders, and 8-hourly rectal washouts. It showed multiple dilated loops of small bowel with air–fluid levels, suggesting small bowel obstruction or ileus (Fig. S2).

**Figure S3.**
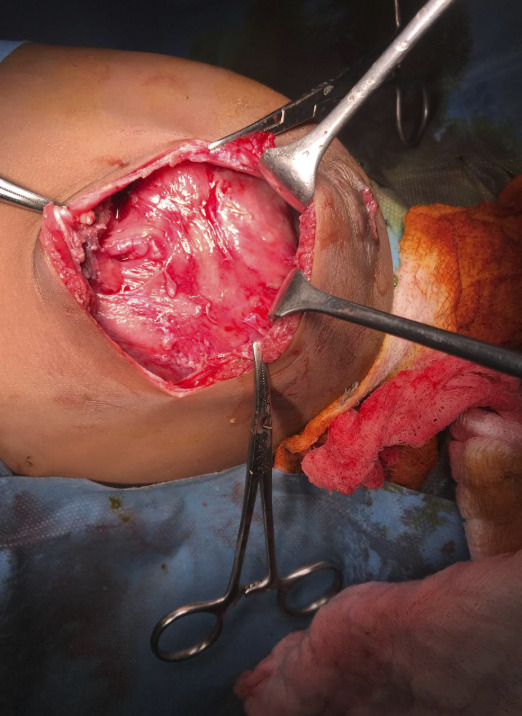
Frozen abdomen.

**Figure S4.**
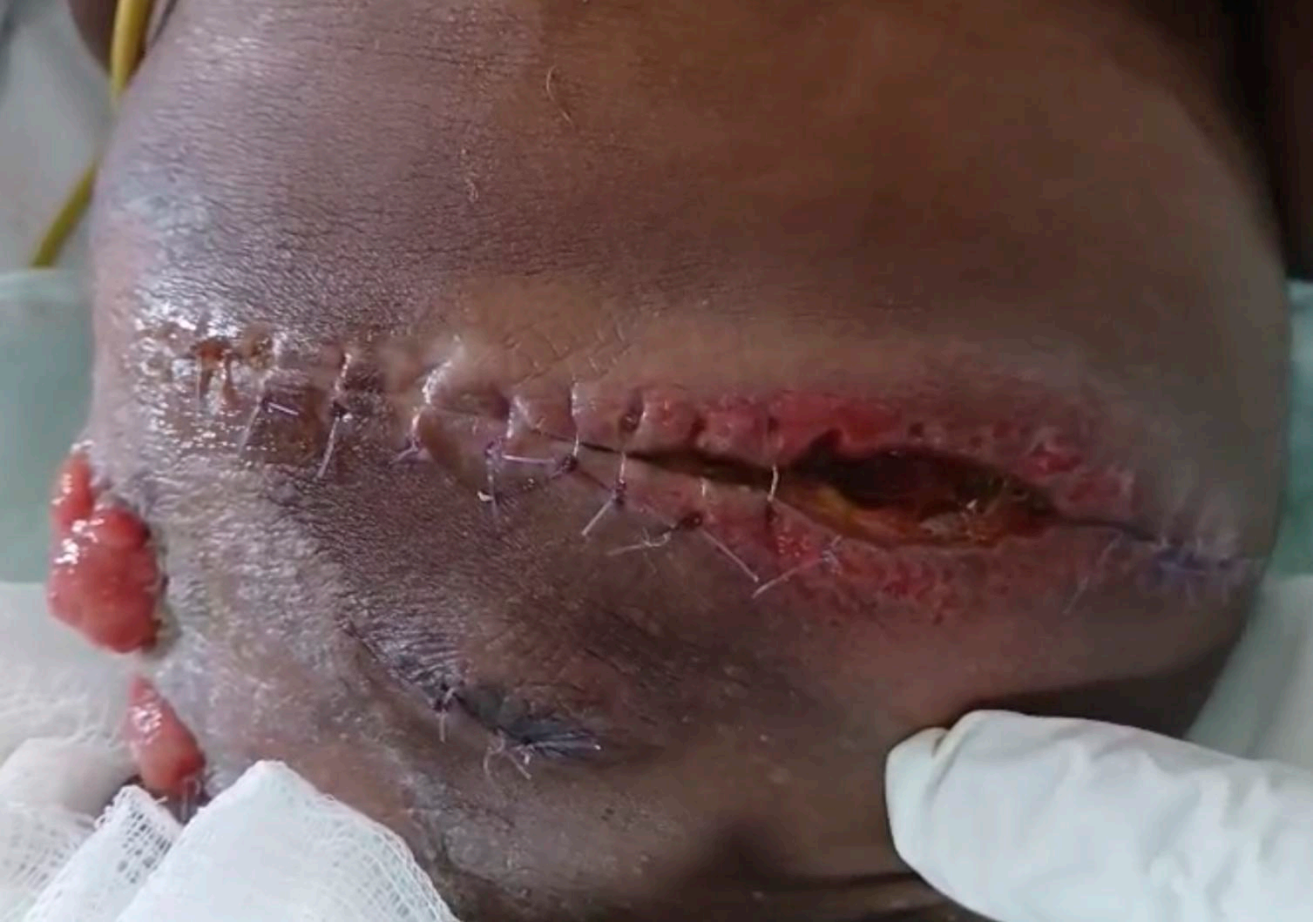
High-output enterocutaneous fistula.

Surgery performed the same day the repeat x ray was done revealed a severely matted small bowel with frozen abdomen (Fig. S3). Granulomatous lesions with enlarged mesenteric lymph nodes were also noted in the peritoneum. Resection of the perforated gut segment was done with an ileostomy fashioned 150 cm from the duodenojejunal junction. Two weeks after surgery, the child developed a high-output enterocutaneous fistula, draining fecal matter (Fig. S4).

**Figure S5.**
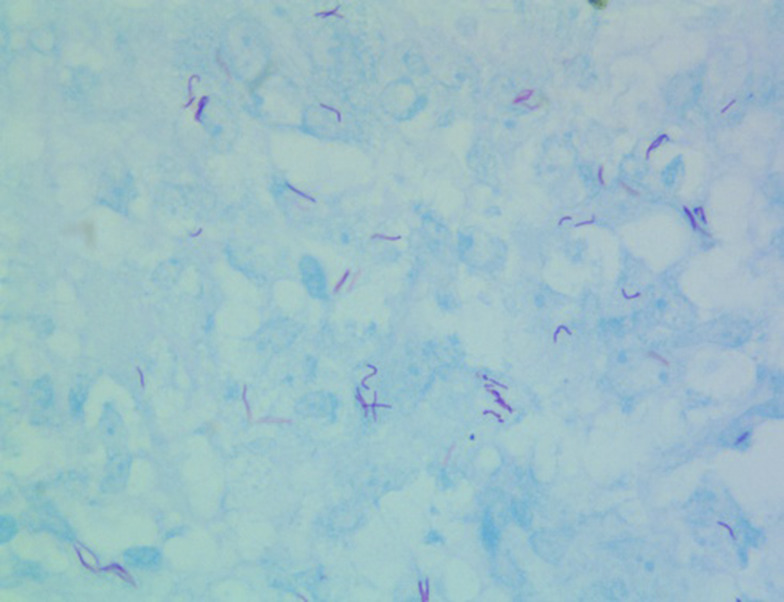
Histology of the small bowel wall.

An intra-abdominal pus swab culture grew multi–drug-resistant *Klebsiella pneumoniae*. Histology of the small bowel wall revealed mucosal ulcerations of the entire wall, which together with the Peyer’s patches bore a necrotizing granulomatous inflammation with epithelioid- and Langerhans-type giant cells and numerous acid-alcohol fast *Mycobacterium* bacilli (Fig. S5). A specimen of granulomatous bowel tissue tested TB PCR (MTB/RIF) negative.

The child was initially treated with piperacillin–tazobactam (40 mg/kg/dose every 8 h), metronidazole (7.5 mg/kg/dose every 8 h), and gentamicin (7.5 mg/kg/dose once daily) due to the initial diagnosis of sepsis with an intra-abdominal focus. First-line once-daily oral anti-TB drugs comprising 1 tablet of the fixed-dose combination of rifampicin (75 mg), isoniazid (50 mg), pyrazinamide (150 mg), 1 tablet of ethambutol (100 mg), and pyridoxine (12.5 mg) were commenced upon confirming the diagnosis of intestinal TB. On day 10 of oral anti-TB, it was noted that all the medication was being excreted through the high-output stoma. A decision was made to change to a modified parenteral holding antibiotic regimen with activity against mycobacteria consisting of linezolid, amikacin, and ciprofloxacin. After 10 days, linezolid was substituted with amoxicillin–clavulanic acid due to the onset of pancytopenia (Table 1).

Persistent fever eventually settled after 20 days of treatment, but the continuous high-output effluent from the enterocutaneous fistulae and the surgical stoma contributed significantly to malnutrition and electrolyte imbalance. The child did not tolerate enteral feeds, and TPN was initiated alongside the wholly parenteral treatment regimen. During the course of treatment, his hepatic impairment related to TPN progressed to liver failure with refractory thrombocytopenia, coagulation abnormalities, and bleeding from the stoma. He eventually succumbed to the extensive complications and died after 2 mo of treatment. Mycobacterial culture and sensitivity results obtained 2 wk after the patient’s demise isolated *Mycobacterium Tuberculosis Complex* sensitive to rifampicin, isoniazid, ethambutol, and streptomycin. Postmortem was not done as the relatives did not consent to the procedure.

## Discussion

Abdominal TB is one of the uncommon forms of extrapulmonary TB comprising ∼3% of all TB cases (10, 11). When there is GIT involvement, treatment can be particularly challenging if there is a major compromise of the absorptive intestinal lining, precluding the use of oral anti-TB medication.

The first hurdle encountered in this case was the difficulty in establishing a diagnosis of TB due to the unavailability of a detailed case management history of the deceased mother. The absence of prominent respiratory symptoms in the child added to the diagnostic challenges. Childhood TB is usually underdiagnosed as the symptoms vary widely requiring the consolidation of various clinical parameters and high index of suspicion from the clinician as outlined in the desk guide developed for primary healthcare workers (12).

The youngest case of intestinal TB that we found in reported literature was 18 mo old. This patient had both enterocutaneous and ileovesical fistulae, requiring complex surgery. Liu et al. (13) in their case report, however, did not mention the extent of gut resection or whether the anti-TB drugs were administered orally or parenterally. Another surprising finding was the absence of acid-alcohol fast bacilli in the resected granulomatous tissue sent for histology. In contrast to other reported cases of intestinal TB, the patient made a quick recovery and was discharged within 2 wk. Two out of the ten abdominal TB cases documented by Boukthir and colleagues from Tunisia (14) had intestinal TB. They were older children aged 11 and 13 years. Their immunity and nutritional status were not documented, and neither is there a record of the extent of intestinal disease. They both had favorable outcomes following standard anti-TB treatment, but there was no report of any surgical procedures having been done.

The onset of symptoms shortly after birth makes congenital TB a possibility in our case. This form of TB is transmitted transplacentally or by aspiration of infected amniotic fluid in the maternal genital tract during delivery. The onset of symptoms ranges from days to weeks, but intervals as long as 128 days have been reported (15). In the systematic review conducted in 2019 by Chaofeng et al. (16), the mortality was 43%, with delayed diagnosis and rapid progression being the chief contributors to poor outcomes. The fact that the child in our case report in addition to the likelihood of congenital TB had severe immunosuppression from a probable inborn error of immunity may have contributed to the rapid progression of TB with the poor outcomes. This finding points to the need for early diagnosis of immunodeficiency. The combination of flow cytometry and immunoglobulin profile tests for demonstrating lymphocyte subsets and immunoglobulin counts, respectively, can serve as initial screening tests for immunodeficiency in patients presenting with severe or unusual presentations of infections. Low T, B, and NK lymphocytes, as well as low immunoglobulin counts—particularly IgM, which is not transferred transplacentally like IgG—should raise the suspicion of severe immunodeficiency conditions (17). Our patient had low lymphocyte subsets, but the immunoglobulin profile did not meet the criteria for immunosuppression. This discrepancy in results can be explained by the presence of maternally transferred IgA and IgG or immunodeficiency subtypes that are characterized by raised IgM such as Omenn syndrome (18). Functional lymphocyte assays and genetic testing though not widely accessible in most healthcare facilities in low-resourced settings should be done to confirm the diagnosis and commence targeted management of the underlying immunodeficiency.

The child had concomitant sepsis and metabolic instability at the initial presentation to our facility, and surgery was therefore deferred for a week. The delay in timely diagnosis resulted in extensive damage to the small intestine with perforations and adhesions leading to a frozen abdomen with the corrective surgical procedures proving unsuccessful in restoring effective gut function. It is also possible that the initiation of anti-TB therapy caused healing by cicatrization worsening the intestinal function. The cicatrization phenomenon was documented as one of the complications of TB treatment among 36 immunosuppressed patients and was thought to be related to the TB-related immune reconstitution inflammatory syndrome (19). Intestinal failure necessitated the use of TPN and medication supplemented by intravenous fluids to balance the excessive losses from the intestinal stoma and enterocutaneous fistulae.

The WHO childhood TB guidelines do not provide guidance for parenteral TB therapy. We adapted our holding anti-TB regimen from published case reports, noting that they also did not have a consensus. Additionally, we consulted national and international TB experts, consolidating their recommendations with literature findings to come up with a regimen that incorporated drugs available locally.

We were severely limited in our medication options by these factors: one limitation was the unavailability of parenteral formulations of any of the conventional TB drugs locally. The antibiotics we selected were from the reserve options outlined by the WHO antimicrobial use guidelines, implying that they carried a risk of fueling antimicrobial resistance. Additionally, these drugs are costly and, being broad-spectrum antibiotics, exposed the patient to the risk of serious side-effects including invasive candidemia.

Another emergent challenge was azotemia and worsening of the hepatic function, emanating from the prolonged use of TPN. Jeejeebhoy (20) in his article on the management of parenteral nutrition–induced cholestasis concludes that in the setting of sepsis and short bowel syndrome, mortality from parenteral nutrition–induced cholestasis is high and such patients should be considered for combined small bowel/liver transplantation if feasible. In our case, the patient had the combination of short bowel and sepsis, leading to eventual multi-organ failure and death.

## Materials and methods

A 3-mo-and-20-day-old infant was referred to our facility with a gradual onset of abdominal distension that began soon after birth and was associated with frequent, foul-smelling, nonbloody stools. One week prior to admission, he developed bilious vomiting and high fever, accompanied by cough.

This baby, the last born of 4 children whose ages were not disclosed but were reportedly alive and well, was born at term with a birth weight of 2.1 kg and had no complications reported during the perinatal period. Antenatal records were unavailable, and therefore, the HIV status of the mother was unknown. She died 1 mo after delivery, following an ailment described as cough with abdominal swelling and weight loss over a number of weeks, with no clear diagnosis. The child was exclusively breastfed for 2 wk with formula milk being introduced at the time of the mother’s hospitalization. His immunization history was unknown, and he exhibited delayed motor milestones with poor neck support by 3 mo of age. He was also noted to have had poor weight gain since birth.

The relatives sought initial treatment from a traditional healer who administered herbal remedies for “teething syndrome.” Failure to respond to this treatment led to admission at the nearby hospital with eventual referral to our hospital for more specialized care. There was no medical report detailing the treatment from the referring facility.

**Figure S6.**
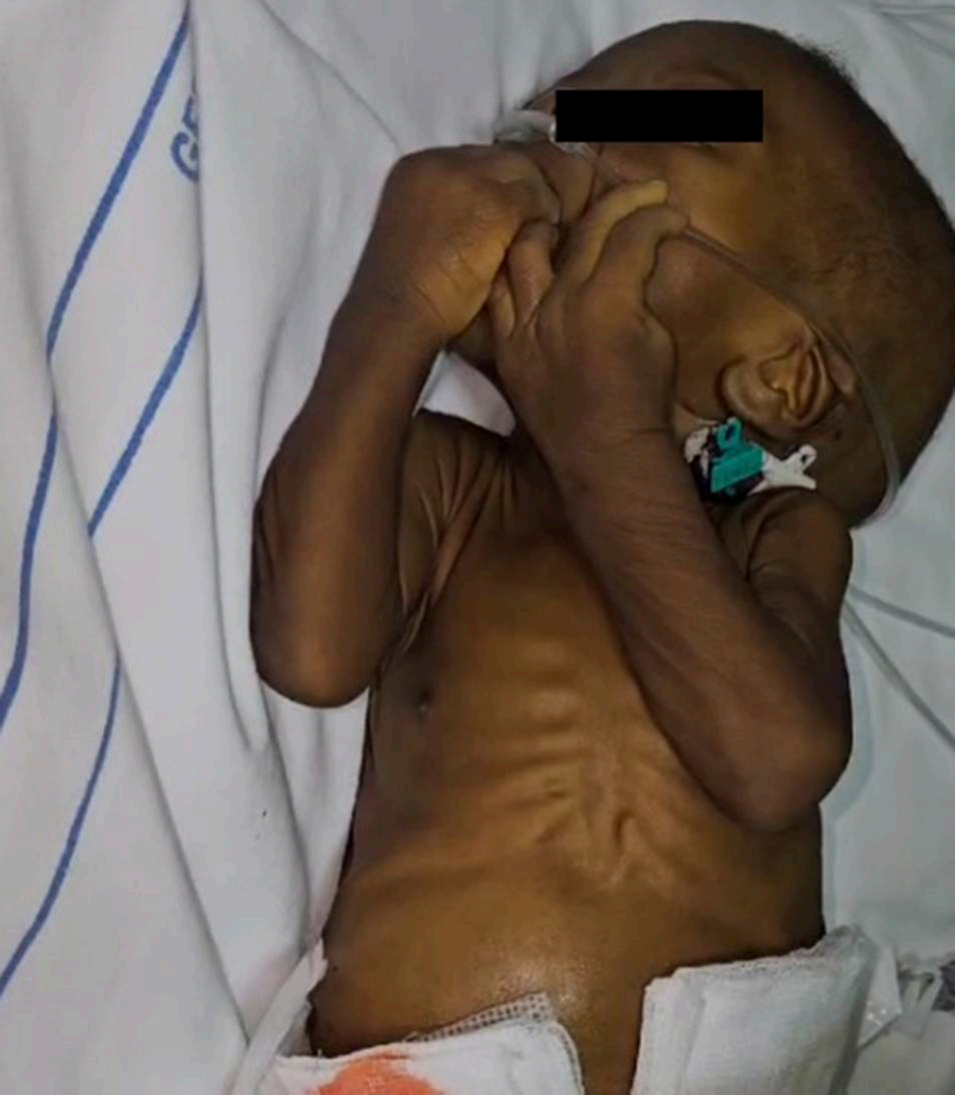
Wasted infant with abdominal distension.

Physical examination at admission revealed an irritable, visibly wasted infant, weighing 4.9 kg and a length of 60 cm, which translated to a z-score of -2SD weight for length. He was febrile, in respiratory distress with tachypnea and low oxygen saturation (SPO2 88% on room air). Further examination showed florid oral candidiasis and a distended, tense abdomen (Fig. S6).

### Conclusion

This case demonstrates the need to consider TB diagnosis in different clinical conditions in high-burden TB settings. There is a need to develop guidelines for parenteral TB treatment. Additionally, in unusual presentations of TB, the clinician should investigate for immunodeficiency.

### Online supplemental material

Figs. S1, S2, S3, S4, S5, and S6 consist of photographic images in JPEG (.jpg) format illustrating clinical, radiologic, and histologic findings relevant to the study. Fig. S1 shows wasted infant with abdominal distension. Fig. S2 shows inflamed bowel loops with gaseous distension. Fig. S3 shows dilated bowel loops with air–fluid levels. Fig. S4 shows frozen abdomen. Fig. S5 shows high-output enterocutaneous fistula. Fig. S6 shows histology of the small bowel wall.

## Data Availability

Data are available in the article itself and its supplementary materials.

## References

[1] World Health Organisation . 2024. Global Tuberculosis Report 2024, xxx, Geneva.

[2] World Health Organisation . 2024. WHO Consolidated Guidelines on Tuberculosis. World Health Organisation, Geneva.

[3] Barker, R.D. 2012. Clinical tuberculosis. Medicine. 40:340–345. 10.1016/j.mpmed.2012.03.002

[4] Debi, U., V. Ravisankar, K.K. Prasad, S.K. Sinha, and A.K. Sharma. 2014. Abdominal tuberculosis of the gastrointestinal tract: Revisited. World J. Gastroenterol. 20:14831–14840. 10.3748/wjg.v20.i40.14831 25356043 PMC4209546

[5] Ha, H.K., G.Y. Ko, E.S. Yu, K. Yoon, W.S. Hong, H.R. Kim, H.Y. Jung, S.K. Yang, K.N. Jee, Y.I. Min, and Y.H. Auh. 1999. Intestinal tuberculosis with abdominal complications: Radiologic and pathologic features. Abdom. Imaging. 24:32–38. 10.1007/s002619900436 9933670

[6] Petrosyan, M., and R.J. Mason. 2006. Tuberculous enteritis presenting as small-bowel obstruction. Clin. Gastroenterol. Hepatol. 4:xxiii. 10.1016/s1542-3565(05)00857-8 16469667

[7] Wang, J., and D. Micic. 2021. Hepatobiliary manifestations of short bowel syndrome and intestinal failure-associated liver disease. Clin. Liver Dis. (Hoboken). 17:297–300. 10.1002/cld.1053 33968392 PMC8087921

[8] Goldani, L.Z., C.O. Spessatto, D.L. Nunes, J.G. Oliveira, E. Takamatu, C.T. Cerski, and H.A.S. Goldani. 2015. Management of severe gastrointestinal tuberculosis with injectable antituberculous drugs. Trop. Med. Health. 43:191–194. 10.2149/tmh.2015-09 26543395 PMC4593777

[9] Rawat, A., K. Arora, J. Shandilya, P. Vignesh, D. Suri, G. Kaur, R. Rikhi, V. Joshi, J. Das, B. Mathew, and S. Singh. 2019. Flow cytometry for diagnosis of primary immune deficiencies-A tertiary center experience from North India. Front. Immunol. 10:2111. 10.3389/fimmu.2019.02111 31572360 PMC6749021

[10] Sharma, S.K., and A. Mohan. 2004. Extrapulmonary tuberculosis. Indian J. Med. Res. 120:316–353. 15520485

[11] Graham, S.M., and J.N. Oliwa. 2023. Diagnosis and Management of Tuberculosis in Children and Adolescents. A Desk Guide for Primary Health Care Workers. The Union, Paris, France.

[12] Sheer, T.A., and W.J. Coyle. 2003. Gastrointestinal tuberculosis. Curr. Gastroenterol. Rep. 5:273–278. 10.1007/s11894-003-0063-1 12864956

[13] Liu, G., T. Chen, X. Song, B. Chen, and Q. Kang. 2023. Case report: A case report and literature analysis on intestinal tuberculosis intestinal perforation complicated by umbilical intestinal fistula and bladder ileal fistula. BMC Infect. Dis. 23:559. 10.1186/s12879-023-08550-z 37641023 PMC10464473

[14] Schaaf, H.S., A. Bekker, and H. Rabie. 2023. Perinatal tuberculosis—An approach to an under-recognized diagnosis. Front. Public Health. 11:1239734. 10.3389/fpubh.2023.1239734 38026389 PMC10661895

[15] Boukthir, S.M.S., S. Mrad, F. Becher, S. Khaldi, and S. Barsaoui. 2004. Abdominal tuberculosis in children: Report of 10 cases. Acta Gastroenterol. Belg. 67:245–249. 15587330

[16] Chaofeng, L., L. Liu, and Y. Tao. 2019. Diagnosis and treatment of congenital tuberculosis: A systematic review of 92 cases. Orphanet J. Rare Dis. 14:131. 10.1186/s13023-019-1101-x 31182120 PMC6558871

[17] van der Burg, M., and A.R. Gennery. 2011. Educational paper. The expanding clinical and immunological spectrum of severe combined immunodeficiency. Eur. J. Pediatr. 170:561–571. 10.1007/s00431-011-1452-3 21479529 PMC3078321

[18] Hsu, C.-C., J.Y.-Y. Lee, and S.-C. Chao. 2011. Omenn syndrome: A case report and review of literature. Dermatol. Sin. 29:50–54. 10.1016/j.dsi.2011.05.002

[19] Amoura, A., T. Frapard, X. Treton, L. Surgers, L. Beaugerie, M. Lafaurie, J.M. Gornet, R. Lepeule, A. Amiot, E. Canouï, . 2024. Tuberculosis and immune reconstitution inflammatory syndrome in patients with inflammatory bowel disease and anti-TNFα treatment: Insights from a French multicenter study and systematic literature review with emphasis on paradoxical anti-TNFα resumption. Open Forum Infect. Dis. 11:ofae327. 10.1093/ofid/ofae327 38957691 PMC11218776

[20] Jeejeebhoy, K.N. 2005. Management of PN-Induced Cholestasis. *In* Nutrition Issues in Gastroenterology. Vol. 24. Practical Gastroenterology, Westhampton Beach. 62–68.

